# Therapeutic Notes

**Published:** 1901-07

**Authors:** Nelson W. Wilson

**Affiliations:** Buffalo, N. Y.


### Progress in Medical Science.

### THERAPEUTIC NOTES.

EDITED BY NELSON W. WILSON, M. D., Buffalo, N. Y.

PEL'S POWDER FOR GASTRIC ACIDITY.

PROFESSOR PEL, of Amsterdam, after a great deal of experience, has found that the following powder is a most excellent remedy for gastric acidity:

| | | | |
|---|---|---|---|
| ℞ | Bicarbonate of soda . . . . . . . . . . . | 10.00 | ʒiiss. |
| | Calcined magnesia . . . . . . . . . . . . . | 8.00 | ʒii. |
| | Bromid of soda . . . . . . . . . . . | 10.00 | ʒiiss. |
| | Carbonate of bismuth . . . . . . . . . . . | 5.00 | gr. lxxv. |
| | Sugar of milk . . . . . . . . . . . . . . . | 10.00 | ʒ ijss. |
| | Oil of fennel . . . . . . . . . . . . . . . | .26 | gtt. iv. |

Mix. Dose, from half to a whole teaspoonful; to be taken an hour after eating. An extra dose in case of pain.

---

### FOR HEMORRHOIDS.

THE *New York Medical Journal*, which is famous for the excellence of its therapeutic notes, gives the following for hemorrhoids:

| | | | |
|---|---|---|---|
| ℞ | Fluid extract of æsculus hippocastanum . . . | 32.00 | ℥i. |
| | Chloroform . . . . . . . . . . . . | 4.00 | ʒi. |

Mix. Morning and evening, at meal times, 10 to 15 drops are to be taken in a glass of wine or sweetened water.

| | | | |
|---|---|---|---|
| ℞ | Fluid extract æsculus hippocastanum . . . . | 24.00 | ʒii. |
| | Fluid extract of hamamelis . . . . . . . . . | 10.00 | ʒiiss. |
| | Oil of peppermint . . . . . . . . . | .13 | gtt. ii. |

Mix. Fifteen drops of this mixture at meal times, morning and evening, in wine or sweetened water.

---

### TO WARD OFF URETHRAL CHILLS.

THIS annoying result of internal urethrotomy or the passing of sounds may be warded off by using Weir's prescription, which follows:

| | | | |
|---|---|---|---|
| ℞ | Sulphate of morphine . . . . . . . . . . | .006 | gr. $\frac{1}{10}$ |
| | Tincture of aconite . . . . . . . . . . . | .13 | m. ii. |
| | Oil of wintergreen . . . . . . . . . . . . | 1.00 | m. xv. |

Mix. For one dose.

---

### THE ADMINISTRATION OF DORMIOL.

*Merck's Archives* gives the following methods for the administration of dormiol, the new hypnotic compound of chloral and amylene hydrate:

| | | | |
|---|---|---|---|
| ℞ | Dormiol, 10 per cent. solution . . . . . . . | 105.00 | ℥iiiss. |
| | Raspberry syrup sufficient to make . . . | 120.00 | ℥iv. |

Mix. Dose, one or two tablespoonfuls at bedtime.

| | | | |
|---|---|---|---|
| ℞ | Dormiol, 10 per cent. solution . . . . . . | 90.00 | ℥iii. |
| | Extract of licorice . . . . . . . . . . . . | 4.00 | ʒi. |
| | Syrup sufficient to make . . . . . . . . . . | 120.00 | ℥iv. |

Mix. Shake label. Dose, a tablespoonful at bedtime.

### FOR AMENORRHEA.

THE *Jour. de Med. de Bordeaux* says Huchard's treatment for amenorrhea is as follows: He directs that the lumbar region be rubbed on retiring with this mixture:

| | | | |
|---|---|---|---|
| ℞ | Chloroform | 10.00 | ʒ ii. ss. |
| | Ether | 20.00 | ʒ v. |
| | Spirits of camphor | 90.00 | ℥ iii. |

Supplement this by hot mustard foot baths, mustard to the inner aspects of the thighs and vaginal injections of water from 105° to 120° F. As a tonic he orders:

| | | | |
|---|---|---|---|
| ℞ | Iron and potassium tritrate | 6.00 | ʒiss. |
| | Socotrine aloes | 1.00 | gr. xv. |
| | Extract of absinth | 2.00 | gr. xxx. |
| | Extract of artemisia | 2.00 | gr. xxx. |
| | Oil of anise | .10 | gtt. iii. |

Mix and make into fifty (50) pills.

Dose—Two after each meal.

---

### AN INTESTINAL ANTISEPTIC.

A VERY good intestinal antiseptic is this which is credited by the *Med. News* to the *Jour. de Med. de Bordeaux*:

| | | | |
|---|---|---|---|
| ℞ | Betanapthol | 3.00 | gr. xlv. |
| | Chloroform | .03 | gtt. xv. |
| | Castor oil | 90.00 | ℥ iii. |
| | Spirit of peppermint | .02 | gtt. v. |

Mix. Adult Dose: A tablespoonful in port wine, beer or sweetened black coffee. Child's dose: A teaspoonful.

---

### FOR WHOOPING COUGH.

THE *Journal of the American Medical Association* says that Dr. R. A. Lancaster has had great success in treating whooping cough with this mixture:

| | | | |
|---|---|---|---|
| ℞ | Tincture belladonna | 16.00–24.00 | ʒiv.–vi. |
| | Whiskey | 30.00 | ℥i. |
| | Phenacetine | 10.00 | ʒiiss. |
| | Fluid extract chestnut leaves | 180.00 | ℥vi. |

Mix. Shake. Label.

Dose—From ten (10) drops for a child one year old to a teaspoonful for a 10-year old child every two to six hours.

